# The Need for Businesslike Methods

**Published:** 1916-11-18

**Authors:** 


### NATIONAL INSURANCE ACT DEVELOPMENTS.

### The Need for Businesslike Methods.

National Health Insurance, though conducted under Government control, is essentially a business enterprise. But it is difficult to imagine anything less businesslike than the manner in which National Health Insurance matters are being treated at the present time by the responsible officials.

Apart from the fact that there are State subsidies, and that for certain large sections of the population the insurance is compulsory, the scheme should be very little different from what it would be if conducted by a mutual insurance society or even by a profit-making company. The insured persons are, in fact, the clients who pay their contributions for certain stipulated benefits, and the approved societies are the business organisations with which, on behalf of the Government, the insurance contracts are made. In a commercial sense the insured have a right to demand that the conditions of their insurances should be made as simple and as easily understood as it is possible to make them, and, moreover, it should be possible for them to judge whether it is likely that the societies to which they are paying their contributions will be able to pay the benefits, which are held out in prospect, when called upon to do so. The approved societies, too, which in a sense are the agents of the Government for the practical administration of the insurance scheme, have a right to expect that the conditions under which they conduct their business shall be free from unnecessary details, particularly during war-time with their staffs depleted. The societies ought, further, to be assured that the terms upon which they are called upon to transact their business, which terms are not of their own making, but are regulated by Act of Parliament, are capable of being carried out.

### Urgent Questions.

Why is it, then, we may fairly ask, that when it has been shown by a strong Departmental Committee appointed by the Treasury that the financial basis of the system requires alteration, the necessary steps for its amendment are so long delayed? Why is it that this same Committee, appointed so long ago as February last, and whose interim report, published in May, made reference to their desire to expedite the completion of their labours, have not yet published their final report on the amendments in administrative detail which experience has shown to be desirable? Are the amendments so numerous, or so difficult to frame? Or is National Insurance once again to be the plaything of party politics?

If in any ordinary business enterprise it were clearly demonstrated that the operations were being conducted at a loss, or that certain important departments were not paying and could not be made to pay without substantial alteration, the directors would immediately take appropriate steps to rectify the position. Delay would only mean added loss, and promptness of action would be the first consideration. Why is it not so with National Insurance? Does the fact that the business is controlled by the Government put it on a different plane from a commercial enterprise? These are important questions, and they are becoming, if they have not already become, urgent.

### Unexplained Delay and its Consequences.

It is doubtless true that the war is absorbing the attention and demanding the energies of Cabinet Ministers, but this is no reason why National Insurance matters should be allowed to drift. The details of National Insurance never have been and never can be thoroughly mastered by each member of the Government. These details are, and must be even at their best, of too intricate a nature to be thoroughly apprehended except by the responsible Minister and his experts, and their services are still available in spite of the war. The war cannot, therefore, be regarded as a valid reason for delaying necessary reforms. On the contrary, it has introduced conditions which make reform all the more necessary and urgent, for it is obvious to anyone who has had any practical experience of the administration of National Health Insurance that there is ample opportunity for simplifying the system without causing injustice. In these circumstances it becomes increasingly difficult to understand why needed business reforms are not carried out in a businesslike way.

The delay which has already occurred has probably made it more difficult to make amendments long overdue than it would have been if they had been introduced earlier in the year, and it is to be feared that further delay will only add to the difficulties. There is a special reason for this, arising from the fact that the Government are only prepared for the present to introduce legislation when it is of a non-contentious character. Legislation there must be if the needed reforms are to be rendered possible, and when the Departmental Committee presented their interim report in May last it seemed as though an Amendment Bill based upon their recommendations would receive general approval. The scheme proposed was admittedly tentative, but it would have had the advantage of securing the immediate object—namely, the prevention of the disclosure of a large number of deficiencies on the first valuation. Moreover, it was an integral part of the Committee's proposals that the scheme should be reconsidered when the Act had been ten years in operation, and by that time there was every reason to suppose that the accumulated experience would form a substantial basis upon which to rear the financial scheme in a final and permanent form.

### An Unofficial Investigation.

Unfortunately, as it now seems, one member of the Departmental Committee, Mr. P. Rockliff, who took a more pessimistic view of the present position than did his colleagues, was unable to sign the interim report. In his view it was absolutely necessary that further State subsidies should be made in order to prevent a large number of approved societies from falling into a condition of insolvency, or rather to obviate the necessity of reducing benefits. His report could not be accepted by the Treasury, as his recommendations exceeded the terms of reference. In consequence of this he communicated his recommendations to the press and resigned from the Committee.

The immediate outcome of this disagreement was the appointment by the Faculty of Insurance of a Commission of Investigation, with Mr. John Hodge, M.P., as chairman, and Mr. Handel Booth, M.P., and Mr. G. W. Currie, M.P., among others, as members. Although this Commission of Investigation is quite unofficial, it has attracted a considerable amount of attention owing to the fact that as certain prominent members of Parliament are on the Commission, its meetings have been held in one of the committee-rooms of the House of Commons. Without the restriction of official terms of reference, but with instructions to review the whole position of National Insurance, this unofficial Commission of Investigation is much less hampered than the official Departmental Committee of Inquiry. It has, in fact, been able to bring within its purview not only matters relating to the administration by approved societies of sickness, disablement, and maternity benefits, to which the official Committee are restricted, but it has also considered matters relating to the administration by Insurance Committees of medical and sanatorium benefits.

In some respects the enlarged scope of the investigation is an undoubted advantage, but from some other points of view it is open to question whether it would not have been more politic for the Faculty of Insurance to have supported the official inquiry by endeavouring to secure the maximum of administrative reform within the limits prescribed by the terms of reference.

### Opposition to the Committee's Recommendations.

It is now clear, however, that the Commission of Investigation intends to raise objection to the recommendations of the Departmental Committee, as set forth in their interim report, if and when they are embodied in an Amendment Bill for the sanction of Parliament. This is made evident by a letter recently addressed by Mr. John Hodge, as chairman of the Commission, to trade union approved societies, in which he says: "We ought to insist upon a full investigation, and not have the matter covered up for the next ten years by a Departmental Committee plaster. If this were to be allowed, the present bad position of trade union approved societies will ultimately be made worse."

The suggestion here made is that the particular interests of the approved societies formed in connection with various trade unions were not sufficiently considered by the Departmental Committee. We have no means of ascertaining if this were so or not, but it is to be noted that the representatives of the type of approved societies referred to who are members of the Committee signed the report, and it must, therefore, be assumed that they were in agreement with its recommendations.

### The Need for Action.

The point, however, which we wish to emphasise is that the unbusinesslike delay which has occurred in bringing the labours of the Departmental Inquiry to a conclusion has resulted in the creation of an opposition which, for the time being, it was most desirable to avoid. If there were so many administrative reforms to be considered it should surely have been foreseen that it would have been better to have deferred the publication of the principal recommendations as to the financial basis of the scheme. It would probably have been better still if the Committee had determined to limit their recommendations on administrative detail to those most obviously requiring reform. Thus, for instance, the conditions regulating the insurance of women, with their present complications of options on marriage and special benefits on re-entry into insurance or widowhood, and the present cumbersome system of dealing with arrears, might have been considered without taking into account other minor matters. If this had been done it should surely have been possible for the report to have been submitted in time for the introduction of the necessary legislation before the adjournment. As matters now stand we merely have the interim report, and the legislation arising therefrom appears as far off as ever. Not one of the approved societies really knows at the present time how its finances stand. They have been conducting the business on varying regulations now for upwards of four years, and throughout the whole of that time it has been known that the margin between success and failure has been slender. The societies ought to know where they stand. If the Government do not intend to press forward at an early date with their proposals for reform, which they virtually admitted to be necessary by the appointment of the Departmental Committee, they should act frankly and order the first valuations to be made as contemplated by the Act of 1911.

### Insurance Commissioners should Act as Business Men.

The special object of providing in the original Act for the valuation of the assets and liabilities at frequent intervals was to ensure that the operations of the societies should be carried out on a sound financial basis, and to provide a corrective for lax administration. Is it to prevent a rude awakening to the nation that the valuations have been postponed? If so, it seems a curious procedure. It is not only the insured persons and the societies in which they are insured who have an interest in the matter. The nation as a whole is interested, financially and socially. The State subsidies are provided from taxes, and taxpayers have a right to know that their money is being spent economically and to the best advantage; while the prevention and cure of sickness among the insured population should be reflected in better conditions of life, even among those who are not insured.

